# Rheological Behavior of High Cell Density *Pseudomonas putida* LS46 Cultures during Production of Medium Chain Length Polyhydroxyalkanoate (PHA) Polymers

**DOI:** 10.3390/bioengineering6040093

**Published:** 2019-10-09

**Authors:** Warren Blunt, Marc Gaugler, Christophe Collet, Richard Sparling, Daniel J. Gapes, David B. Levin, Nazim Cicek

**Affiliations:** 1 Department of Biosystems Engineering, University of Manitoba, Winnipeg, MB R3T 5V6, Canada; David.Levin@umanitoba.ca (D.B.L.); Nazim.Cicek@umanitoba.ca (N.C.); 2 Scion Research, Te Papa Tipu Innovation Park, 49 Sala Street, Private Bag 3020, Rotorua 3046, New Zealand; Marc.Gaugler@scionresearch.com (M.G.); Christophe.Collet@scionresearch.com (C.C.); Daniel.Gapes@scionresearch.com (D.J.G.); 3 Department of Microbiology, University of Manitoba, Winnipeg, MB R3T 2N2, Canada; Richard.Sparling@umanitoba.ca

**Keywords:** PHA, viscosity, non-Newtonian fluid, fed-batch fermentation, oxygen transfer, *Pseudomonas putida*

## Abstract

The rheology of high-cell density (HCD) cultures is an important parameter for its impact on mixing and sparging, process scale-up, and downstream unit operations in bioprocess development. In this work, time-dependent rheological properties of HCD *Pseudomonas putida* LS46 cultures were monitored for microbial polyhydroxyalkanoate (PHA) production. As the cell density of the fed-batch cultivation increased (0 to 25 g·L^−1^ cell dry mass, CDM), the apparent viscosity increased nearly nine-fold throughout the fed-batch process. The medium behaved as a nearly Newtonian fluid at lower cell densities, and became increasingly shear-thinning as the cell density increased. However, shear-thickening behavior was observed at shearing rates of approximately 75 rad·s^−1^ or higher, and its onset increased with viscosity of the sample. The supernatant, which contained up to 9 g·L^−1^ soluble organic material, contributed more to the observed viscosity effect than did the presence of cells. Owing to this behavior, the oxygen transfer performance of the bioreactor, for otherwise constant operating conditions, was reduced by 50% over the cultivation time. This study has shown that the dynamic rheology of HCD cultures is an important engineering parameter that may impact the final outcome in PHA cultivations. Understanding and anticipating this behavior and its biochemical origins could be important for improving overall productivity, yield, process scalability, and the efficacy of downstream processing unit operations.

## 1. Introduction

Recent concern over the accumulation of plastic waste in the natural environment (particularly micro-plastics) emphasizes the need to find alternative biodegradable polymers [1,2]. In this regard, PHA polymers are a promising replacement for petroleum-based plastic materials, being both renewable and completely biodegradable [3]. PHA polymers can have a variety of different monomer sub-unit compositions. This enables, to a large extent, a wide-range of physical and thermal properties and numerous potential applications [4,5]. Indeed, certain PHAs (depending on the composition and arrangement of the monomer subunits) have properties comparable to conventional petroleum-based plastics, like polyethylene and polypropylene.

PHA polymers are synthesized as intracellular reserves of carbon, energy, and reducing power by a wide-range of bacteria, and some archaea. While the cost of production currently limits applications for PHA to niche markets [6,7], development of more efficient bioprocesses may help to increase the economic viability and lessen the environmental impact of PHA production [8,9]. Currently, HCD cultures are widely seen as the best cultivation strategy to achieve high volumetric productivities [10]. Some HCD cultures for PHA production have reached cell densities in excess of 200 g·L^−1^ CDM [11,12,13]. Further details on HCD cultivations in PHA production are available in several recent reviews [14,15]. However, a common problem with HCD cultures in general is increasing medium viscosity [16]. This can lead to dead zones in the bioreactor and reduced heat and mass transfer capabilities, especially in large-scale bioreactors with inherently poor mixing capability [17]. 

Rheology is the study of the deformation of matter (in this case, the flow of liquid fermentation medium) under an applied stress. Previous studies have examined the rheology of cultivation medium for a variety of bioprocessing applications using different microorganisms and fungi. These include: xanthan gum production using *Xanthomonas* spp. [18], viscous mycelial (fungal) cultures for variety of bio-products [19,20,21,22,23,24], polyglutamic acid (PGA) production using *Bacillus subtilis* [25], mixtures of primary and secondary sewage sludge [26], and fermentation of sewage sludge [27,28], amongst others. Multiple studies have looked at rheological properties of extracellular polymeric substances (EPS) produced by *Pseudomonas* spp. [29,30,31,32,33]. However, many of these assessments examined rheological properties of an extracted polymer of interest, but did not directly quantify its effect on culture medium. Most studies show that fermentation medium behaves as a non-Newtonian fluid, meaning the apparent viscosity is dependent on the shear rate [19,23,34,35].

Since lack of adequate dissolved oxygen (DO) is a significant factor that limits productivity in HCD cultivations for PHA production [36], the effect of medium viscosity on the oxygen transfer rate could be important. Several previous studies have demonstrated inversely proportional relationships between viscosity and oxygen transfer in both model Newtonian fluids (glycerol, glucose solutions) as well as non-Newtonian fluids (xanthan gum, carboxymethylcellulose solutions) [17,37,38,39,40,41,42]. Such model fluids are often preferred to actual biological cultures because they are cheaper and easier to work with [17]. A few studies, however, have evaluated oxygen transfer characteristics in a biological medium [19,25]. In all cases, there is a consensus that the volumetric oxygen mass transfer coefficient, *K_L_a*, is inversely proportional to the medium viscosity. 

Previous application of HCD cultivations in PHA production are numerous [15]. Yet, we can find no evidence that rheology of the culture medium has been studied to date; or at the very least, that information is not widely accessible. This includes both short-chain length (scl-) PHAs and medium chain length (mcl-) PHAs. Considering that PHAs are high molecular weight (*M_w_*) polymers that can occupy up to 75–88% CDM [13,43] and be produced with relatively high titer [14,15], the examination of culture rheology and its effects on oxygen transfer could be an important contribution to process development, optimization, and scalability in PHA production. Furthermore, this could have significant impact on downstream processing, including pumping, filtration, centrifugation, or spray drying unit operations. 

The objectives of this work were, therefore: 1) to examine time-dependent rheological behavior of HCD fed-batch cultures of *Pseudomonas putida* LS46 for production of medium chain length (mcl-) PHAs; 2) to gain understanding of the biochemical origins of these rheological changes; and 3) to further assess how viscosity impacts the oxygen mass transfer characteristics of the cultivation medium. 

## 2. Materials and Methods

### 2.1. Micro-Organism, Medium, and Substrate

The strain used in this study was *Pseudomonas putida* LS46 [44], and strain maintenance procedures were as specified previously [45]. A slightly modified version of Ramsay’s minimal medium used in all experimental studies [46]. However, the initial concentrations of (NH_4_)_2_SO_4_, MgSO_4_, CaCl_2_·2H_2_O, and trace element solution were increased to 2 g·L^−1^, 0.2 g·L^−1^, 20 mg·L^−1^, and 2 mL·L^−1^, respectively. The MgSO_4_, CaCl_2_·2H_2_O, ferric ammonium citrate, and trace element solution were filter sterilized through a 0.2 μm filter after autoclaving. Octanoic acid was used as the substrate in these studies and was added through a sterile 0.2 μm filter after autoclaving to an initial concentration of 20 mM. 

### 2.2. Reactor Setup and Operation

Most experiments for this work were conducted in a 7 L (total volume) bench-scale system with a 3 L working volume. This system was used to generate the meta-data supporting the rheological observations in the pilot-scale bioreactor, which is described below. The configuration and setup of the bench-scale bioreactor system has been described previously [45,47]. Aeration was maintained at a constant flow rate of 2 VVM (atmospheric air only), and a mixing cascade (350–1200 rpm) was used to control the DO signal at 40% (of saturation with atmospheric air at 30 °C) for as long as possible. A reactive pulse-feed strategy was applied in response to either a drop in the off-gas CO_2_ signal or a rise in the DO signal, indicating carbon limitation. Sub-inhibitory pulses of octanoic acid (5–20 mM) and a 200 g·L^−1^ solution of (NH_4_)_2_SO_4_ were added to the reactor via high-precision injector syringes automated by LabBoss software [48]. The bench-scale cultivation was performed three times. 

Because of the larger sample volume (1 L) required for rheological analysis, the system used for generation of these samples was a pilot-scale stainless steel, sterilization in place (SIP) bioreactor with a 152 L total volume (Sartorius Stedim Biostat D-DCU, Göttingen, Germany). The bioreactor was equipped with three 160 mm diameter Rushton turbines, four baffles, pH and DO electrodes, and a ring-type sparger located underneath the impeller. The bioreactor was filled with an initial volume of 70 L medium, and sterilized at 121 °C for 20 min before cooling to 30 °C. 

In the pilot-scale system, the DO was maintained at 40% (of air saturation at 30 °C) for as long as possible. The cascade for DO was maintained through: (1) incremental increases in pressure from 200 mbar to 1000 mbar; (2) incremental increases in stirring rate from 100 rpm to a maximum of 600 rpm; and (3) increasing aeration (atmospheric air only) from 10 litres per minute (LPM) up to 30 LPM (maximum of approx. 0.4 volumes of air per liquid volume per minute or VVM). At this scale, aeration was limited because of foaming and excessive gas holdup encountered at higher volumetric flow rates. The slight headspace overpressure was used to obtain similar growth rates and biomass production over time, as well as timing of the onset of oxygen-limited conditions, as compared to the bench-scale bioreactor. This implies less efficient mixing in the pilot-scale bioreactor. The pilot-scale experiment was also carried out using the above-described pulse-feed strategy, except feeding was done with calibrated peristaltic pumps. Due to time and resource constraints, the pilot scale cultivation was performed once. 

In either bioreactor system, experiments were initiated with the addition of a 5% (vol/vol) inoculum, which was grown overnight in flask cultures. After 16–20 h, (NH_4_)_2_SO_4_ was no longer fed because it was no longer being consumed rapidly due to DO limitation. The pH of the medium was generally maintained via the addition of NaOH with automated peristaltic pumps (4 M at bench-scale and 10 M at the pilot-scale). 

### 2.3. Sample Treatment

Samples (20–40 mL) were periodically withdrawn from the bioreactor, generally in 1–3 h intervals. These were centrifuged for 10 min at 12,500× *g*. The pellet was washed once in PBS buffer, transferred into a pre-weighed 20 mL aluminum dish and dried at 60 °C until no further loss of mass was detected to determine the total biomass concentration ([*X_t_*], g·L^−1^ CDM). The PHA content of the biomass (*%_PHA_*) was determined by gas chromatography with a flame ionization detector (GC-FID) using the sample preparation, instrument, and operating parameters described previously [45]. The supernatant was decanted and stored at −20 °C for analysis of residual octanoic acid by GC-FID and ammonium was determined spectrophotometrically by the indophenol blue method. Further details of these analyses are available elsewhere [45]. 

### 2.4. Viscosity Measurements (Pilot Scale)

Periodic 1 L samples were withdrawn from the bioreactor for rheological analysis. For certain samples, a portion of the medium was centrifuged at 12,500× *g* for 15 min (Sorvall RC-6 Plus with an F12-6 × 500 LEX rotor) to investigate the cell-free supernatants. The medium viscosity was assessed using a DHR-2 Rheometer (TA Instruments, New Castle, DE, USA) equipped with a cup-and-bob measurement system (30 mm cup diameter; 28 mm bob diameter). The cup and bob geometry was chosen to mitigate effects from sample drying, but plate-plate and cup/vane geometry were also assessed. Although good results for all three measurement geometries were obtained, the cup/bob system was chosen because of a more defined flow in the measurement gap, lower end-effects compared to the vane geometry [49] and fewer artefacts due to sample drying during the test compared to the parallel plate geometry. 

The samples were conditioned at 30 °C for 20 min prior to measuring. During this conditioning step, a constant shear of 1 s^−1^ was applied to avoid settlement of the samples. All samples were measured using a flow sweep between 2 and 1000 s^−1^. The samples in the cup/bob assembly were inspected after completion of the rheological testing to ensure that no significant evaporation occurred that would have affected the viscosity results. All samples were measured in triplicate. The data analysis was done using TRIOS v4.1.0.31739 (TA Instruments, New Castle, DE, USA). 

The measured shear stress at different shear rates during rotational rheology can be described using a variety of established rheological models. In this work, the fit was best described using the power law. A power-law fluid is an idealized fluid, and its shear stress is a function of shear rate as described by

(1) $$\tau =\varphi \times {\dot{\gamma }}^{n}$$

where *τ* is the shear stress (mPa); *φ* is the Power law viscosity constant (mPa·s); ${\dot{\gamma }}^{}$ is the shear rate (s^−1^), and *n* is the rate index (dimensionless). 

### 2.5. Off-Line Measurement of the Volumetric Oxygen Mass Transfer Coefficient (Bench-Scale)

The global volumetric oxygen mass transfer coefficient, *K_L_a*, was measured using the dynamic out-gassing method [50]. To avoid the impracticality of *K_L_a* determinations at scale (which would require a 3 L sample volume), a small-scale reactor with a 200 mL working volume was constructed to allow at-line *K_L_a* determination while using minimal (150 mL) sample volume taken at various points throughout the bench scale fed-batch cultivations. The goal was to show that, for a given reactor environment (with constant mixing, geometry, gas flow rates, etc.) the oxygen transfer performance of that system is reduced as the chemical matrix of the supernatant becomes increasingly complex and viscous over time. An unfortunate consequence or limitation, however, is that the determined *K_L_a* values are not representative of the actual reactor environment from which the samples were derived. Because of this, the results were expressed as a percent of the value measured using the 0 h sample. 

This 200 mL reactor used for *K_L_a* determination was constructed from plexi-glass with height 7.9 cm and 5.4 cm in diameter. The reactor was equipped with compression fitting ports for a DO probe, gas inlet, and gas outlet. The reactor was stirred magnetically with a 2.5 cm stir bar at 1000 rpm, and either air or N_2_ was delivered to the reactor at flow rates of 200 or 500 mL·min^−1^, respectively. This was done using thermal mass flow controllers (Bronkhorst Hi-Tech, Ruurlo, the Netherlands), which were part of an off-gas sensor system previously described [48]. A minimum of three determinations was done for each sample, and this was replicated for three fed batch experiments. The unit was validated initially in trials using distilled water or Ramsay’s medium, and *K_L_a* values of 34.3 ± 3.4 h^−1^ and 21.5 ± 2.1 h^−1^ were obtained, respectively. Not surprisingly, these were on the lower end of the values obtained previously in the bench-scale bioreactor system [45,47]. This is probably because: (1) lack of baffles in the miniature device; (2) a stir bar was used instead of a proper impeller in the miniature device; and (3) the point of release of the bubbles was above the stir bar in the miniaturized reactor (as opposed to underneath the impeller in the bioreactor).

### 2.6. Analysis of Organic Products in the Supernatant (Bench-Scale)

Soluble protein in the supernatant was determined spectrophotometrically at 595 nm using a modified Bradford Assay [51]. Briefly, 0.5 mL of supernatant was mixed with 0.5 mL of 0.4 M NaOH. The samples were boiled for 10 min, and centrifuged (12,500× *g* for 5 min). A 20 μL aliquot of each sample was then placed in triplicate wells of a 96-well plate with 200 μL of Bradford Reagent (obtained from Sigma-Aldrich, St Louis, MO, USA). Standards were prepared using bovine serum albumin (Sigma-Aldrich, St Louis, MO, USA) and diluted into 0.2 M NaOH at concentrations of 0–300 mg·L^−1^. Samples outside this concentration range were diluted appropriately in distilled water and the analysis was redone. 

Reducing sugars in the supernatant were determined by the Anthrone method adapted from a previous protocol [52]. Briefly, 0.5 mL of supernatant was added to glass reaction vials with sealed caps. Then 1 mL of 0.1% anthrone in concentrated H_2_SO_4_ was added to the vial (using filter tips) and sealed. Samples were placed in a water bath at 80 °C for 5 min, and then allowed to cool to room temperature. 200 μL of each sample was pipetted (again using filter tips) into triplicate wells in a 96-well plate and the color change (green-blue) was quantified spectrophotometrically at 620 nm. Standards were prepared using glucose at concentrations of 0–100 mg·L^−1^. 

DNA in the supernatant was quantified using the Qubit Fluorometer. A 5 to 20 μL volume of sample was diluted into a 200 μL total volume of the working solution and sample (working solution was the fluorescence dye diluted 1:200 in buffer). If further dilutions were required, the samples were diluted in distilled water. The broad range DNA standards were used, which had a concentration range of 0 to 5 ng·μL^−1^. 

Volatile solids in the supernatant were quantified using 50 mL crucibles. A known volume of supernatant was placed in pre-weighed crucibles that were kept in a desiccator. The crucibles were then oven-dried at 105 °C for 24 h and weighed again following an equilibration period in the desiccator, and then placed at 550 °C for at least 2 h. The final mass of the crucible was then measured following cooling and equilibration in a desiccator. 

## 3. Results and Discussion

### 3.1. Growth and mcl-PHA Synthesis

**Figure 1:**
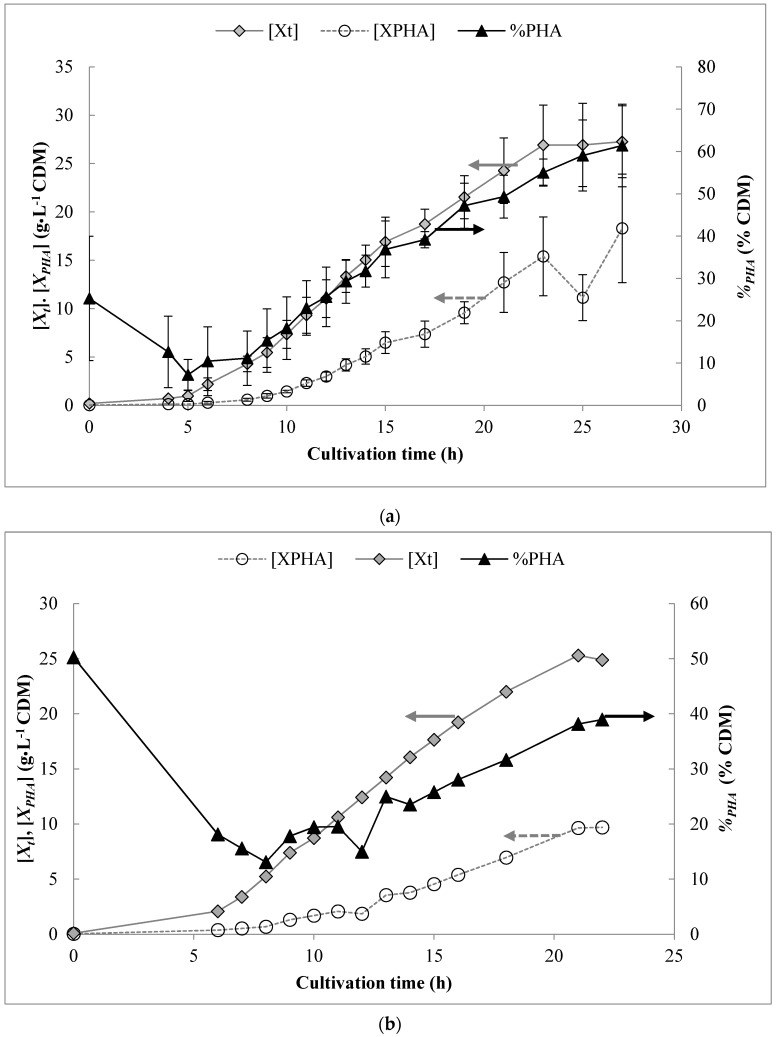
Results for biomass and PHA production obtained over the course of the pulse-feed fed-batch experiments (**a**) bench-scale bioreactor system (3 L initial working volume) and (**b**) pilot-scale bioreactor system (70 L initial working volume).

The [*X_t_*], *%_PHA_*, and resulting PHA biomass ([*X_PHA_*], expressed in g·L^−1^) are shown in Figure 1 for both bioreactor systems. The initially high mcl-PHA content at time zero is due to carry-over from the inoculum, which was grown in flasks for a sufficiently long period so as to induce oxygen limitation and mcl-PHA synthesis from octanoic acid [45]. At both scales, the onset of oxygen limitation occurred around 12–14 h post inoculation and caused carbon flux to shift from growth to mcl-PHA synthesis. Overall, growth and total biomass production were similar at both scales, although the final *%_PHA_* in the pilot-scale bioreactor was slightly lower than at bench-scale. 

### 3.2. Rheological Characterization of the Cultivation Medium

**Figure 2:**
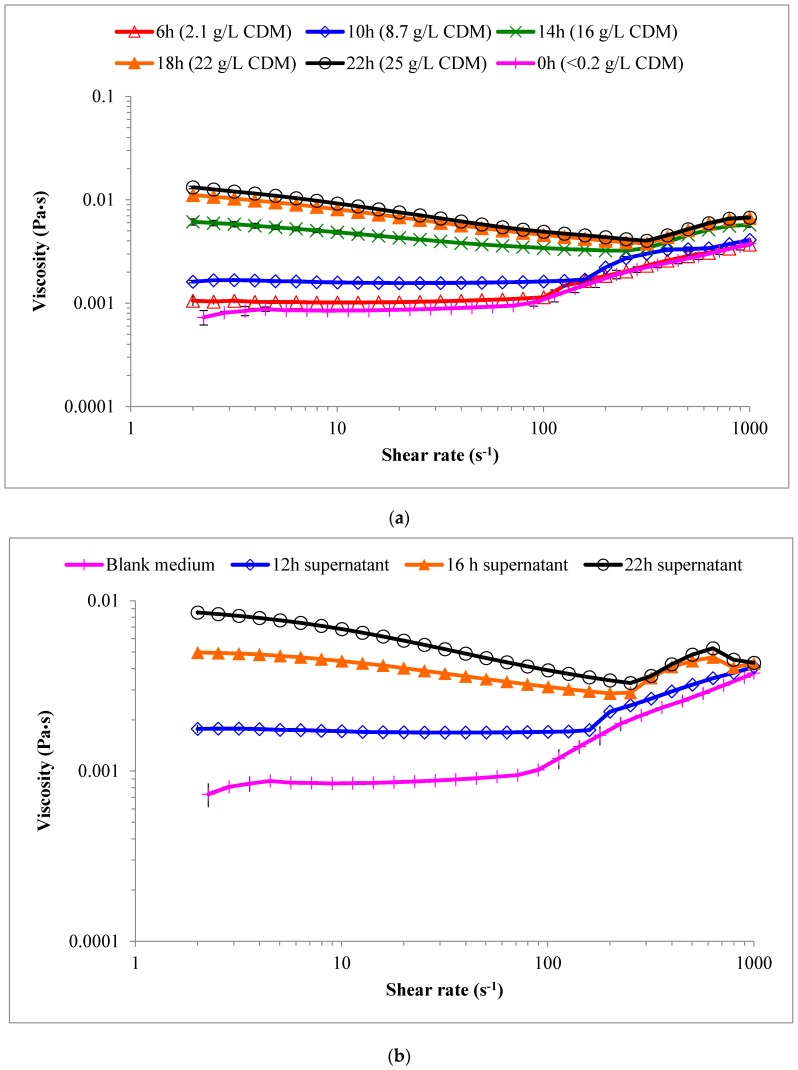
Flow sweep curves for samples of *P. putida* LS46 cultures obtained from the pilot-scale reactor. (**a**) Viscosity as a function of shear rate for 0–25 g·L^−1^ cell suspensions at various points in time and (**b**) viscosity as a function of shear rate for supernatant samples at various points in time. Error bars represent standard deviations of triplicate measurements for each sample.

Over the time course of the cultivation, the medium (even after centrifugation) became increasingly opaque and viscous. This appeared to significantly dampen the turbulence created for a given stirring input. The flow sweep curves for samples obtained from the pilot-scale system show viscosity as a function of shear rate for both cell suspensions (Figure 2a) and cell-free supernatants (Figure 2b) at various points in time in the bioreactor. In both cases, the medium appeared to behave as a Newtonian fluid up until 12 h for shear rates of approximately 75 s^−1^ or less. By 14 h (which corresponded to the onset of O_2_ limitation in the bioreactor), a significant increase in viscosity was observed and the samples also became increasingly shear-thinning. 

This type of behavior has been described in previous studies of a variety of fermentation processes [34,35,53]. Similar rates were used in this study, but a unique attribute of this work is the viscosity of samples began to increase at a certain shear rate, which was consistent across the different measurements for the individual samples and increased with increasing sample viscosity (and hence cultivation time). This indicates a material property-related root cause rather than a measurement artefact. 

In the initial (0 h) sample, this shear-thickening behavior was observed at shear rate of 76.5 s^−1^, and increased with increasing sample viscosity up to nearly 300 s^-1^ in the final sample (25.3 g·L^−1^ CDM). This is an interesting observation because similar shear rates can easily be encountered in a bioreactor. This could suggest that increasing the shearing rate (bioreactor agitation rate) beyond this shear-thickening onset may actually cause a viscosity increase and reduce the oxygen transfer rate since viscosity is generally inversely proportional [41]. 

**Figure 3:**
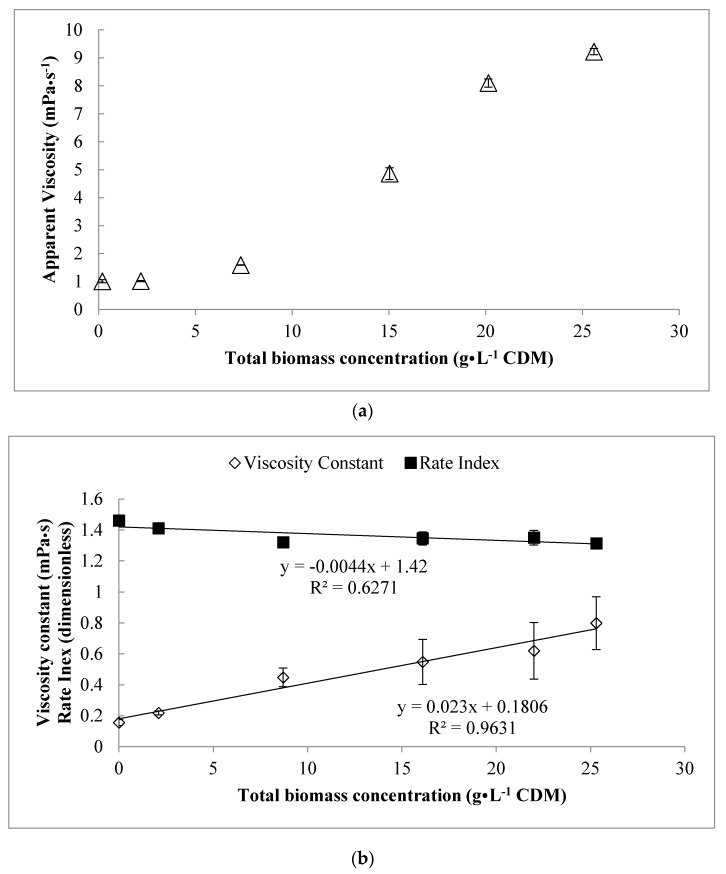
(**a**) Changes in apparent viscosity of the *P. putida* LS46 culture with increasing total biomass concentration over time and (**b**) changes in power-law constants describing culture rheology as a function of the total biomass in the (pilot-scale) fed batch cultivation at varying points over time. Error bars represent the standard deviations between technical replicate measurements (*n* = 3).

The relationship between shear-stress and shear rate was best described using the Power law (*R^2^* > 0.99 for all samples). A summary of model parameters for fitting the data from each sample with the power law is shown in Table 1, and in Figure 3 as a function of the corresponding total biomass of the sample. As shown, a strong linear relationship between the viscosity constant and the total biomass could be derived (*R^2^* = 0.96), and the slope was significantly different than zero (*p* = 0.028). However, the rate index did not seem to correlate with biomass in the culture (*R^2^* = 0.63), and the slope was not significantly different than zero (*p* = 0.27). 

The increase in viscosity of the culture as s function of [*X_t_*] is shown in Figure 3a. The viscosity at a shear rate of 10 s^−1^ was determined and compared from the flow sweep results to quantify the viscosity of the samples. Using a Tukey’s range test, statistically significant (95%) differences in the rotational viscosities and shear-thickening onset across the different samples could be identified. These values are shown in Table 2, where it can be seen that over time, the apparent viscosity of the cell suspension (at a shear rate of 10 s^−1^) increased from approximately 1.0 mPa·s to 9.2 mPa·s by 22 h. Interestingly, when the cells were removed by centrifugation, it was found that the viscosity of the 22 h supernatant was 6.8 mPa·s, which is nearly 75% of the value observed for the entire culture at 22 h (i.e., with [*X_t_*] = 25.3 g·L^−1^) suspended in that same matrix). From observations during the course of this work, when cells from 25.3 g·L^−1^ culture of *P. putida* LS46 (22 h) were re-suspended in fresh medium, the viscosity dropped to 1.7 mPa·s at shear rates of 10 s^−1^, which was only slightly higher than the 0 h sample (1.01 mPa·s).

Typically, electrolyte solutions like microbial growth medium are slightly shear thinning [54]. In colloidal dispersions, shear-thinning is thought to be due to a more organized flow pattern of the molecules when subject to shear forces. This creates less stochastic (random) interactions, and results in reduced viscosity and decreased energy dissipation [55,56]. At higher shear rates, however, hydrodynamic forces can dominate over stochastic interactions, and the particle collisions are primarily due to shear forces rather than random thermal motions. This causes organization of the molecules into a more anisotropic state of so-called ‘hydroclusters’, and increases the difficulty by which molecules can flow around one another [55]. Although other theories exist (including order-disorder transition and dilatancy), this is perhaps the most commonly accepted mechanism for shear-thickening [56]. At the molecular level, the mechanism remains the subject of some debate, all theories essentially pertain to increased difficulty with particle-particle interactions in a flow path, and thus the volume fraction of particles is of importance [56,57]. The presence of high *M_w_* polymers (particularly when suspended in a poor solvent), could further support the shear-thickening observations in this work [58]. In such situations, higher shear rates tend to cause high *M_w_* macromolecules to extend in the flow path, breaking their intra-molecular associations and forming inter-molecular associations. This results in a gel network formation, which increases viscosity [59]. This is also a positive feedback mechanism in which the molecules of higher *M_w_* extend first, and formation of gel networks causes the viscosity to increase. This, in turn, increases the shear stress, which then affects the molecules of lower *M_w_* [59]. The intermolecular associations may include crosslinking, which is a known phenomenon with mcl-PHA [60,61,62,63]. 

### 3.3. Quantifying Components of the Extracellular Matrix

These data indicate that significant rheological changes to the culture medium occur over time, and much of this effect is not simply explained by the presence of cells. According to Newton et al. [53], in HCD *E. coli* cultures this behavior is the result of structural interactions between cells and cellular debris (high *M_w_* nucleic acids, which can also from crosslinks) resulting from lysed cells. This could further contribute to a shear thickening effect. The following section describes the soluble organic material detected in the culture supernatant. 

*P. putida* and other *Pseudomonas* spp. are known to produce significant quantities of extracellular polymeric substances (EPS) as precursors to biofilm formation. This is a particularly well-known phenomena with *P. aeruginosa.* Kachlany et al. [64] suggested that young *P. putida* G7 cells are encapsulated by an exopolysaccharide layer that is sloughed off as cells age. It is likely that high shear forces expedite the sloughing of this capsular material. That study also described the collapsed extracellular polymer from *P. putida* G7 as being a ‘rope-like’ material, which could certainly fit the proposed gel-formation theory for shear thickening behavior in polymer solutions.

Generally, the extracellular polymers associated with *Pseudomonas* spp. are composed predominantly of sugars, typically glucose, galactose, rhamnose and mannose [30,32,64,65,66]. Other extracellular secretions associated with *Pseudomonas* spp. include alginate [67], DNA [68,69,70], gellan [71], proteins [72], glycolipids and lipopolysaccharides [64,73,74], organic acids, [30,75], as well as acetylated sugars and uronic acids [65,66]. 

**Figure 4:**
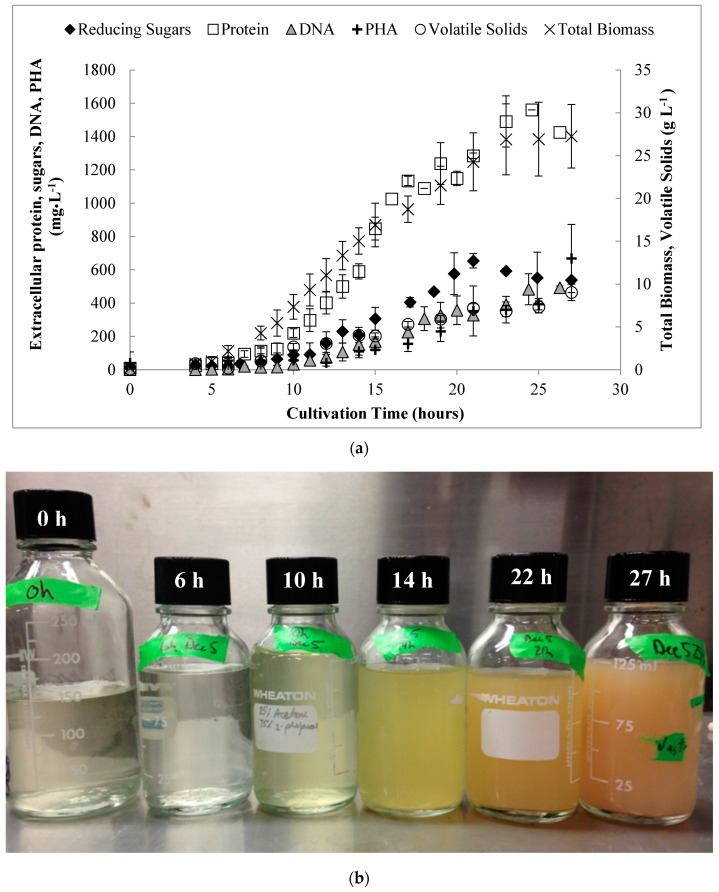
(**a**) Soluble (extracellular) organic material detected in the supernatant over the time course of the bench-scale cultivations, which is thought to contribute to the observed rheological behavior of the medium and (b) appearance of culture after centrifugation for fed-batch experiments at the cultivation time indicated. Error bars represent standard deviations between the mean values obtained from each of the biological replicate experiments.

In this work, several of these putative EPS constituents and/or cell lysis products were monitored and quantified in the supernatants of cultivations performed at bench-scale to better understand the observed rheological behavior. These include proteins, reducing sugars, DNA, and extracellular PHA, as well as bulk measurement of carbonaceous products in the supernatant by volatile solids. These are shown over time in Figure 4. 

In general, the concentrations of these components increased proportionally to [*X_t_*] with the exception of reducing sugars, which reached a maximum concentration of 0.65 g·L^−1^ at 21 h and then declined. The maximum concentrations (at 27 h) of proteins, PHA, and DNA in the medium were 1.56 g·L^−1^, 0.67 g·L^−1^, and 0.49 g·L^−1^, respectively. Collectively, the components could account for at most 48% of the total VS detected in the supernatant, which reached a maximum of 9 g·L^−1^ by 27 h. Newton et al. [35] demonstrated that both protein (0–50 g·L^−1^) and DNA (0–4 g·L^−1^) contributed linearly to increased viscosity and the flow curves for solutions of protein and DNA exhibited shear thinning and Newtonian behavior, respectively. However, in that work a somewhat lesser increase in viscosity was noted (1.1 to 5.3 mPa·s) for a 48 g·L^−1^ *E. coli* culture, despite the presence of far more extracellular DNA (typically 3 g·L^−1^) and protein (up to 40 g·L^−1^). 

In this work, we did not attempt to differentiate whether these components of the supernatant were due to the production of EPS or are simply cell lysis products from cultivation in a high-shear environment. We found evidence to make an argument in favor of either scenario, which likely implies the rheological behavior is due to a combination of physical and biochemical factors. Mg^2+^ is an intracellular metabolite that can leak from damaged cell membranes, which would precede cell lysis [76]. In this work, the Mg^2+^ concentration monitored in the supernatant began to increase after 14 h, which corresponds to the onset of O_2_ limitation and maximum agitation rates. However, when the ratios of protein-to-DNA of the culture supernatant was compared to that of *P. putida* cell lysate, it was found that the cell lysate contained only about half the DNA fraction that was observed in the supernatant. This could suggest DNA release as a possible EPS component, and would be supported by previous studies using *Pseudomonas* spp. [68,72]. Furthermore, a similar experiment using lower shear rates using a maximum of 600 rpm mixing (compared to 1200 rpm) in the bench-scale bioreactor, but with pure O_2_ to increase driving force for oxygen transfer. Although this method produced lower [*X_t_*], statistically indifferent (*p* < 0.05) yields of extracellular organic content (protein, sugars, DNA per unit [*X_t_*]) were observed in comparison with the normal mixing condition of 1200 rpm. While this does not disprove the occurrence of significant cell lysis, it does show that this behavior is difficult to avoid, even at comparatively low bioreactor mixing rates. 

### 3.4. Engineering Significance: Effects on Oxygen Transfer Rate

**Figure 5:**
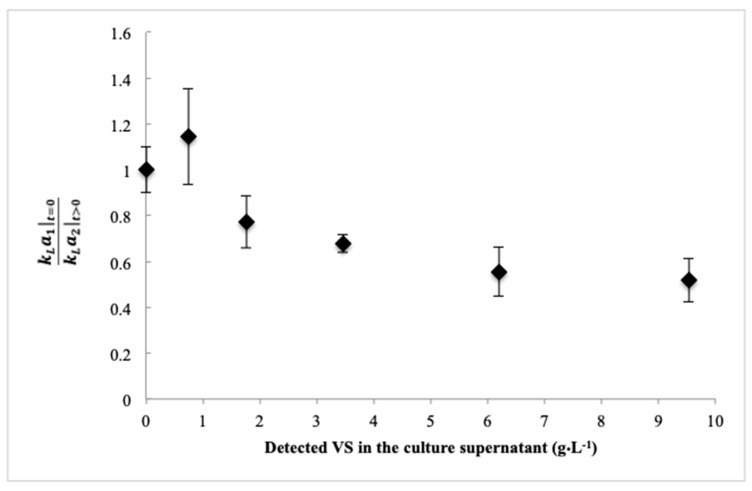
Shows the reduction in *K_L_a* over time (expressed as a fraction of the value measured at time zero) that might be anticipated from the increasing VS detected in the supernatant. The *K_L_a* values were measured in the described 200 mL reactor. Error bars represent standard deviation of the mean values determined for each biological replicate experiments.

Knowledge of viscosity in bioprocesses is important for process scale up. Many empirical relationships (or dimensionless parameters like the Reynold’s number, *Re*) describing the *K_L_a*, are inversely proportional to viscosity [50]. The *K_L_a* measured using the 200 mL bioreactor system with different supernatant samples obtained from the bench-scale bioreactor over time were assessed. The reduction in *K_L_a* over time is shown in Figure 5 as a function of the increasing amount of soluble organic material in the culture supernatant. As shown, *K_L_a* is expressed as a percent of the value measured at 0 h. The final values (obtained at 27 h) showed a significant (*p* < 0.05) reduction of 45–52% from the values measured at 0 h or 6 h. It was intended to perform a similar test using the entire culture, but that was not possible due to the high oxygen demand of the culture preventing observable changes in DO as well as excessive foaming when air was bubbled through the cell suspensions in the miniature reactor. 

According to Martin et al. [41], the reduction in oxygen transfer rates with increased fluid viscosity is due to: (1) reduced contact area between bubbles and a fluid because bubbles are more stable in viscous fluids; and (2) decreased liquid diffusivity due to reduced velocity profile in the liquid layer surrounding the bubble. Interestingly, the extracellular biosurfactants (glycolipids, lipopolysaccharides) known to be secreted by several *Pseudomonas* spp. can form a layer at the liquid–gas interface and decrease oxygen mass transfer [36]. 

Considering that other fed-batch strategies have achieved 100 g·L^−1^ CDM or more in PHA production [11,13,77,78], the reduction in *K_L_a* over time could be considerably more significant in those systems compared to that described in this work. This may often be neglected in bench-scale HCD PHA cultivations in which stirring and aeration are typically set as high as is realistically possible to maximize productivity. However, with increasing scale the required power consumption for mixing and aeration becomes significant [79], and so the bioreactor operation parameters must be carefully managed in order to save aeration costs. This aspect is emphasized in PHA production/waste treatment operations using enriched mixed cultures [80].

A characteristic of shear-thickening fluids is effective energy dissipation [56]. While this may be useful or interesting property for certain applications, for bioprocessing it is generally problematic, and would likely result in poor performance for the energy input to the bioreactor. The Reynold’s number (*Re*) is often used as an dimensionless constant used to estimate power consumption required to mix the reactor contents, which can be a significant cost for aerobic processes like PHA production [79]. Using the obtained constants shown in Table 2, we estimated that for a given (un-gassed) power input to the stirrer, *Re* decreased by 44–55% as the cell density of the culture increased from approximately 0 to 25 g·L^−1^ following the approach of Gabelle et al. [17]. 

Shear thickening behaviour, although interesting, is problematic for the cultivation process as well as downstream operations, including pumping, centrifugation, filtration, or spray-drying [55,56]. In our production process using *P. putida* LS46, it certainly appears to be a difficult situation to avoid. Moving forward, efforts to alleviate such conditions might include: 1) investigation of lower-shear bioreactors such as air-lift configurations [81]; 2) modifying the medium with surface active molecules or flocculants to reduce or reverse shear-thickening [82]; 3) addition of extracellular enzymes to break up large macromolecules that may form gel networks and contribute to increased viscosity and shear-thickening; or 4) or engineering the bacterium to, or selection of strains that, avoid production of EPS. The latter could also help close the carbon balance and improve the overall PHA yield. 

## 4. Conclusions

In moderately HCD fed-batch cultivations of *P. putida* LS46, significant changes in the rheological properties of the culture were observed. At lower shear rates the culture exhibited slight shear-thinning behavior, while the onset of shear-thickening was observed at shear rates that increased with sample viscosity (or increased [*X_t_*]). A nearly nine-fold increase in viscosity (at 10 s^−1^) was measured throughout the course of the cultivation process, approximately 75% of which was attributed to the supernatant rather than the presence of cells. Investigation of the culture supernatants revealed up to 9 g·L^−1^ VS being present in the supernatant, of which half was accounted for as extracellular proteins, sugars, DNA, and PHA. It was shown that this material could reduce the mass transfer coefficient associated with a given bioreactor system by up to 50% over the course of the cultivation process. Although difficulties in maintaining oxygen transfer are well known in HCD aerobic bioprocesses, this work has demonstrated that biochemically-induced changes in the medium composition played a significant role, rather than just the high oxygen demand associated with a HCD culture of strictly aerobic organisms. 

## Figures and Tables

**Table 1:** Summary of model parameters (*n* = 3) for fitting the obtained data from each sample with the power law.

Sample	Power Law Constant: Viscosity (mPa·s)	Power Law Constant: Rate Index	Power Law: Regression
	Mean ± St. Dev.	Mean ± St. Dev.	Mean ± St. Dev.
0 h (<0.2 g·L^−1^)	0.16 ± 0.02	1.46 ± 0.02	1.00 ± 0.00
6 h (2.1 g·L^−1^)	0.22 ± 0.01	1.41 ± 0.01	1.00 ± 0.00
10 h (8.7 g·L^−1^)	0.45 ± 0.06	1.32 ± 0.02	1.00 ± 0.00
14 h (16.1 g·L^−1^)	0.55 ± 0.15	1.35 ± 0.04	1.00 ± 0.00
18 h (22 g·L^−1^)	0.62 ± 0.18	1.35 ± 0.05	1.00 ± 0.00
22 h (25.3 g·L^−1^)	0.80 ± 0.17	1.31 ± 0.03	0.99 ± 0.00
12 h supernatant	0.32 ± 0.02	1.37 ± 0.01	1.00 ± 0.00
16 h supernatant	1.91 ± 0.03	1.12 ± 0.00	0.99 ± 0.02
22 h supernatant	3.15 ± 0.13	1.05 ± 0.01	0.99 ± 0.00

**Table 2:** Summary of average viscosity at a shearing rate of 10 rad·s^−1^ and onset of shear-thickening (*n* = 3).

Sample	Viscosity @ 10 s^−1^, mPa·s	Shear Thickening Onset, s^−1^
	Mean ± St.Dev	Mean ± St.Dev
0 h (<0.2 g·L^−1^)	1.01 ± 0.06	76.5 ± 13.0
6 h (2.1 g·L^−1^)	1.01 ± 0.02	94.8 ± 0.6
10 h (8.7 g·L^−1^)	1.59 ± 0.01	151.1 ± 0.6
14 h (16.1 g·L^−1^)	4.86 ± 0.21	258.9 ± 18.1
18 h (22 g·L^−1^)	8.10 ± 0.15	294.9 ± 9.1
22 h (25.3 g·L^−1^)	9.22 ± 0.11	293.3 ± 12.5
12 h supernatant	1.71 ± 0.01	156.3 ± 2.2
16 h supernatant	4.42 ± 0.07	218.9 ± 2.6
22 h supernatant	6.81 ± 0.09	256.6 ± 8.3
